# Chemical annotation of small and peptide-like molecules at the Protein Data Bank

**DOI:** 10.1093/database/bat079

**Published:** 2013-11-28

**Authors:** Jasmine Y. Young, Zukang Feng, Dimitris Dimitropoulos, Raul Sala, John Westbrook, Marina Zhuravleva, Chenghua Shao, Martha Quesada, Ezra Peisach, Helen M. Berman

**Affiliations:** ^1^Department of Chemistry and Chemical Biology, and Center for Integrative Proteomics Research, Rutgers The State University of New Jersey, 174 Frelinghuysen Rd, Piscataway, NJ 08854-8087, USA and ^2^San Diego Supercomputer Centre and Skaggs School of Pharmacy and Pharmaceutical Sciences, University of California, San Diego, 9500 Gilman Drive, La Jolla, CA 92093-0743, USA

## Abstract

Over the past decade, the number of polymers and their complexes with small molecules in the Protein Data Bank archive (PDB) has continued to increase significantly. To support scientific advancements and ensure the best quality and completeness of the data files over the next 10 years and beyond, the Worldwide PDB partnership that manages the PDB archive is developing a new deposition and annotation system. This system focuses on efficient data capture across all supported experimental methods. The new deposition and annotation system is composed of four major modules that together support all of the processing requirements for a PDB entry. In this article, we describe one such module called the Chemical Component Annotation Tool. This tool uses information from both the Chemical Component Dictionary and Biologically Interesting molecule Reference Dictionary to aid in annotation. Benchmark studies have shown that the Chemical Component Annotation Tool provides significant improvements in processing efficiency and data quality. Database URL: http://wwpdb.org

## Introduction

The Protein Data Bank (PDB) is the international repository for three-dimensional (3D) structures of proteins, nucleic acids and other biologically active molecules. The PDB archive is managed by the Worldwide Protein Data Bank (wwPDB) (1), a collaboration among organizations that act as deposition, processing and distribution centers for PDB data. Structural data are deposited by researchers, and then checked, processed and annotated by biocuration staff at wwPDB member sites [Research Collaboratory for Structural Bioinformatics Protein Data Bank (2), Protein Data Bank in Europe (3) and Protein Data Bank Japan (4)]. To continue to support scientific advancements and ensure the best quality of the data files, the wwPDB partnership is developing a new deposition and annotation (D&A) system (5). This new system is composed of four major modules: the Chemical Component Annotation Tool (CCA Tool) that compares ligand chemistry against the Chemical Component Dictionary (CCD) and the Biologically Interesting molecule Reference Dictionary (BIRD); the Sequence module that reviews the representation of the sequence in the deposited entry, the coordinates and corresponding cross-references in third-party sequence databases (6, 7); the Added Annotation module that calculates biological assemblies, ligand binding sites and secondary structure based on the coordinates and the Validation module (8) that implements wwPDB Validation Task Force recommendations (9–11), including geometry checking of individual polymeric residues and ligands, and the goodness of the fit of the model to the experimental data.

Understanding the interactions between macromolecules and small biologically active molecules is key to deciphering biological function and is critical for drug design and development. Therefore, providing accurate chemical descriptions of these small molecules is a primary focus of PDB annotation.

A typical entry deposited to the PDB archive contains atomic coordinates, polymer sequences, experimental descriptions and structure determination information. PDBx, a format based on the macromolecular Crystallographic Information File, is used to represent macromolecular structure data (12). One of the first steps in annotation is to correctly identify or create the specific ‘chemical components’ that are used in the entry. Chemical components are unique chemical entities of small molecules that appear across the PDB archive, and are defined and cataloged in the CCD. Chemical components are very diverse in nature and include, but are not limited to, ions, solvents, standard and modified amino acids, nucleic acids, antibiotics, inhibitors, metal clusters and surfactants. Each component in the CCD is identified by a unique code (CCD ID), and contains such information as the component’s chemical name, formula, connectivity, bond order, stereochemistry information and chemical descriptors. The CCD definition also includes software-derived ‘idealized’ coordinates that represent the component with molecular connectivity, bond order and chirality that is energetically favorable.

Chemical component annotation is one of the bottlenecks in PDB data processing. The number of PDB depositions containing new ligands has been increasing. For example, ∼2000 entries containing 600 new chemical components were released in the year 2000, as compared with ∼8900 entries containing 1700 new chemical components released in 2012. The complexity of ligands deposited to the PDB archive has also increased over the years, with instances of peptide-like inhibitors and antibiotics, such as vancomycin and thiostrepton, and organometallic complexes. Historically, the annotation of chemical components has involved multiple steps and manual operations.

**Figure 1.**
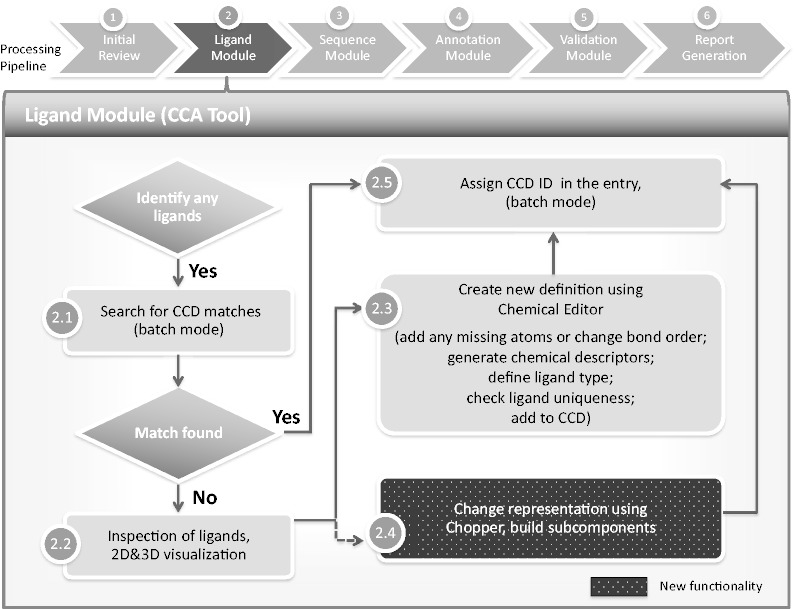
Chemical component annotation. Processing steps are labeled as described in this article. Compared with the previous method of chemical component processing, the CCA Tool automates and integrates most of the steps, including ‘batch’ functionalities that process multiple components at the same time. The CCA Tool is also fully integrated with the D&A system, whereas the previous pipeline was completely separate from the other annotation processes and tools.

The goal of the new CCA Tool is to identify chemical components and capture more accurate chemical descriptions during deposition and to annotate and validate these components more efficiently. Key features include the implementation of automatic ‘batch’ searches of all chemical components from the deposited coordinates against all possible matches in the CCD, and interactive two-dimensional (2D) and 3D comparison views of the deposited coordinates and their closest matches in the CCD. Ligand identification, editing, definition creation and ID assignment have been integrated into the CCA Tool. This tool has been incorporated into current annotation practices at all wwPDB data centers (Figure 1).

## Batch searches against the CCD

As part of chemical component annotation, the CCA Tool automatically identifies all chemical components in a newly deposited entry and locates matching components in the CCD (Figure 1, Step 2.1). The CCA Tool automatically compares the atom types, valencies, connectivities, bond orders and stereochemical identification of the author-deposited coordinates with every entry in the CCD. If simple discrepancies are found, any related explanations or chemical descriptions provided during deposition are reviewed. Authors may be contacted at this point for further clarification and discussion.

**Figure 2.**
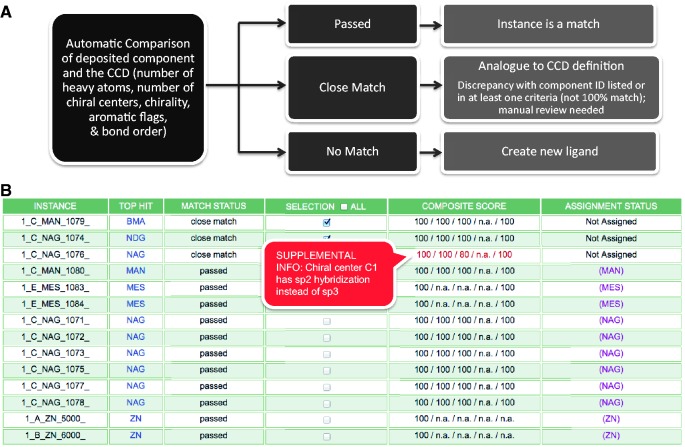
Batch Search Results Report for an entry that contains multiple chemical components (Step 2.1 of Figure 1). (**A**) The CCA Tool identifies and compares deposited ligands with the CCD in a batch mode, and reports the status (passed, close match or no match) of the comparison, which results in corresponding annotator action. (**B**) An example search results report that provides immediate information to the annotator about each chemical component instance found in the entry, and the closest match found in the CCD, as named in the ‘Top Hit’ column. In this example, the deposited entry has 14 chemical components, including two instances of alpha-d-mannose (MAN), eight instances of N-acetyl-beta-d-glucosamine (NAG), two instances of 2-(*N*-morpholino)-ethanesulfonic acid (MES) and two instances of zinc ions (ZN) as listed in the first column. The second column displays closest component matches found in the CCD. The report shown indicates that only the first three instances require further inspection, as the other instances in the deposited entry have corresponding definitions in the CCD. Matches that are similar but not identical can be initially evaluated with the use of the Composite Score column that represents the comparison of the instance with its Top Hit match in five categories: number of heavy atoms, number of chiral centers (independent of handedness), handedness of the chiral centers, number of aromatic atoms and bond order. In each category, each atom in the instance is compared against the Top Hit match in a binary way (match or no match). Then the number of matching atoms is divided by the number of total possible matches (i.e. the number of eligible atoms in the category) and is expressed as a percentage. Mousing over the Composite Score displays additional information in a pop-up window. In this example, the Composite Score does not reveal any chemical differences for the first two instances listed, which means that the only difference between the deposited instance and the top hit match is the CCD ID used. The annotator will then update the CCD ID used in the deposited entry. The third instance listed, 1_C_NAG_1076, has a score of 80% for the chiral center comparison, as one of the five chiral centers in NAG (chiral center C1) has sp2 hybridization rather than sp3 in the experimental coordinates.

The Batch Search Results Report (Figure 2) provides an overview of all chemical components found in a deposited entry. Each occurrence of a particular chemical component from the entry, referred to here as an ‘instance’, is listed in the Results Report with the ‘Top Hit’ component found in the CCD and the corresponding ‘Match Status’. Exact matches between the deposited ligand and the best matching component from the CCD will display a Match Status of ‘passed’. Matches that are similar, but not identical (‘close match’), can be initially evaluated with the use of the ‘Composite Score’ listed in the fifth column. The Composite Score contains five numbers representing the comparison of the instance with its top hit match in five categories: number of heavy atoms, number of chiral centers (independent of handedness), handedness of the chiral centers, number of aromatic atoms and bond order. In each category, every atom in the instance is compared against the Top Hit match in a binary way (match or no match). Then the number of matching atoms is divided by the number of total possible matches (i.e. the number of eligible atoms in the category) and expressed as a percentage. A score of 100% represents an exact match, whereas a lower number represents a less similar comparison in that category. Pop-up windows display additional details about the composite scores to alert the annotator to the geometrical issues found. A status of ‘no match’ indicates that a new chemical component definition needs to be created and added to the dictionary to represent the component.

## Visualization and verification of chemical details and assignment

**Figure 3.**
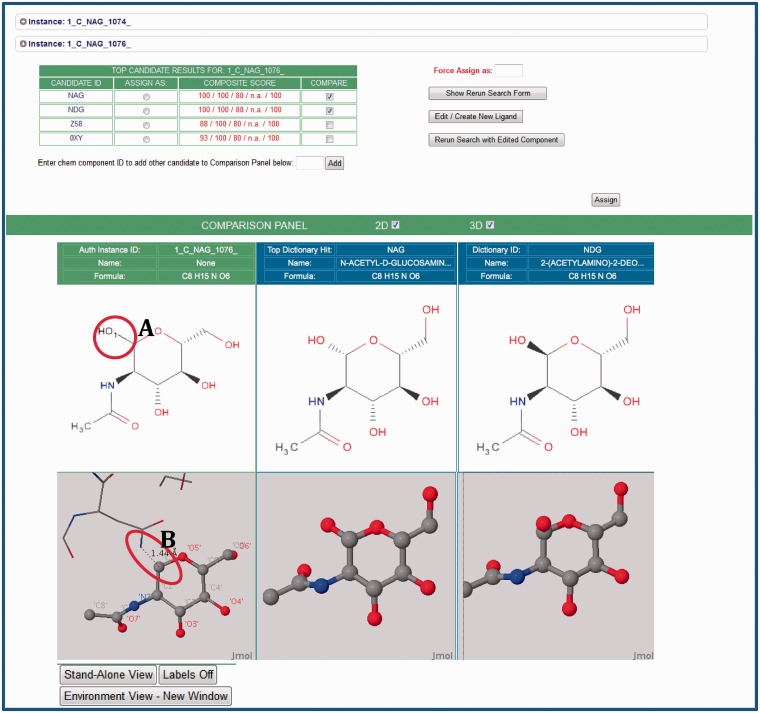
The Instance Search View (Step 2.2 in Figure 1). This example shows two instances in the deposited entry, both labeled NAG (*N*-acetyl-beta-d-glucosamine), that are analogs of the matches found in the CCD. For these instances, the annotator can launch 2D and 3D comparisons by selecting the arrow next to the instance of interest. Visual comparisons of deposited instances (green column) and CCD definitions (blue columns) are available. To suggest CCD matches, the CCA Tool uses the deposited chemical environment for the prediction of the complete chemical description. This environment is displayed as sticks in the visual displays. In this example, the tool has recognized the adjacent atoms, and has added the leaving group as a black stick (labeled A) to provide the absolute stereochemistry. The 3D view reveals the glycosylation interaction of the deposited instance of NAG with asparagine (ASN) (in stick representation, labeled B). The annotator can use this environmental information to correctly assign components.

If an exact match has not been found, the annotator manually selects and further reviews possible CCD matches for each chemical component instance returned (Figure 1; Step 2.2). The Instance Search View (Figure 3) displays a summary of all instances of a particular CCD ID listed in the deposited coordinates. In the example shown in Figure 3, two instances of *N*-acetyl-beta-d-glucosamine (CCD ID NAG) have been identified in the deposited coordinates. The annotator uses this view to examine close matches and other match candidates.

Because small molecules in PDB entries are often covalently bound to a macromolecule (protein or nucleic acid) or to other small molecules, close inspection of the instance within its environment is necessary to identify the correct CCD ID. The CCA Tool takes adjacent connected atoms into account to obtain the complete chemical description of the instance. The 3D environment view allows easy manipulation of the molecule. For example, Figure 3 displays the interaction of NAG 1076 with asparagine (ASN) 219 at the glycosylation site. In this case, the connected atom from the adjacent asparagine 219 (labeled A) was added to atom C1 of NAG 1076 as a leaving hydroxyl (OH1) for the purposes of absolute stereochemical identification of the anomeric carbon (labeled B). In this example, the author-assigned NAG 1076 is a close match to CCD-defined ligands NAG and 2-(acetylamino)-2-deoxy-a-d-glucopyranose (NDG) that differ in the chirality of the C1 atom.

After comparison, the annotator would either indicate that the deposited chemical component instance matches one of the existing CCD definitions or create a new CCD definition.

## Specialized chemical editing features and adding new components

**Figure 4.**
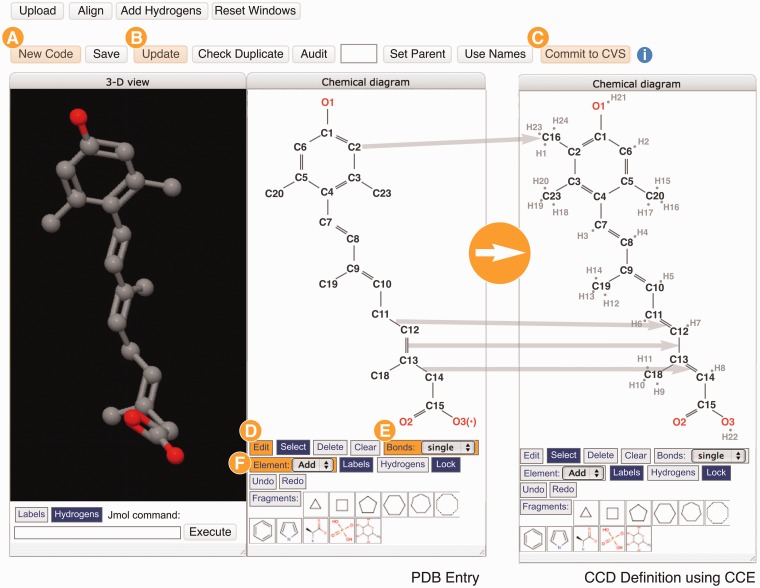
The Chemical Component Editor (Step 2.3 in Figure 1) used to create new CCD definitions. The interface provides a variety of operations (top buttons) to support the creation of a new chemical definition with a unique code (labeled A), update the PDB entry file (labeled B) and add definitions to the CCD (labeled C). Molecular viewers interactively display the chemical component in 2D and 3D. In this example showing the creation of the definition for CCD ID R12, two steps performed are shown in the 2D sketch tool panels: changing bonds from single to double, and from double to single (CCE functions labeled D and E) and then adding missing atoms/elements (labeled F). Hydrogen atoms are added implicitly and the chemical descriptions are updated automatically. Changes are updated instantly in the 2D and 3D viewers.

When an instance in the deposited entry does not match anything in the CCD, the Chemical Component Editor (CCE) is used to define and add the new chemical component to the dictionary (Figure 1, Step 2.3) using the atom types and positions contained within the atomic coordinates. Using the CCE interface (Figure 4), the annotator can obtain a unique CCD ID, edit and save chemical information, update the PDB entry and add this new definition to the CCD. 2D and 3D molecular views, as well as tabular views (not shown), are provided to support the editing operations.

In cases where the atomic coordinates used to derive a chemical component are incomplete or the software-derived bond order is incorrect, the annotator can use the CCE to make changes to the CCD definition such as adding unobserved atoms based on depositor-provided chemical information, changing bond order and interactively generating the idealized coordinates to complete the representation of the molecule. The processed PDB entry lists any missing atoms, but does not include them in the coordinate section. Figure 4 shows the creation of the definition for CCD ID R12 using the CCE. Carbon atom C16 was added to C2, the single bond between C11 and C12 was changed to a double bond and the double bond between C12 and C13 was changed to a single bond. Hydrogen atoms are added implicitly and the chemical descriptions are updated interactively in the table shown.

After the new component has been prepared, a redundancy check against the CCD is performed, and the component is committed to the CCD. The CCD is maintained using Concurrent Versions System. The performance of Concurrent Versions System, particularly for write operations on large data files, outperforms other alternative systems such as Subversion. The CCD is updated weekly as part of the PDB archive. Any updates or changes made to a component after the entry is released are publicly recorded in its CCD definition.

This process is a substantial improvement to the previous system that required manual editing across multiple tools and steps. Chemical component processing time has been significantly reduced, and productivity and accuracy have been improved.

## Representation of complex peptide-like molecules

Along with small molecules, depositions containing pharmaceutically interesting peptides and peptide-like molecules (peptidomimetics) such as complex inhibitors and antibiotics have substantially increased in recent years. Proper annotation of these molecules is critical to the ability to support searching and analysis of the PDB archive. Recently, these molecules were reviewed and the representation and annotation of these molecules remediated and updated for uniformity archive-wide. This remediation resulted in the creation of the new BIRD resource that is similar to the CCD and is used in the annotation of peptide-like molecules with inhibitory/antibiotic properties (http://www.wwpdb.org/bird.html) (13).

**Figure 5.**
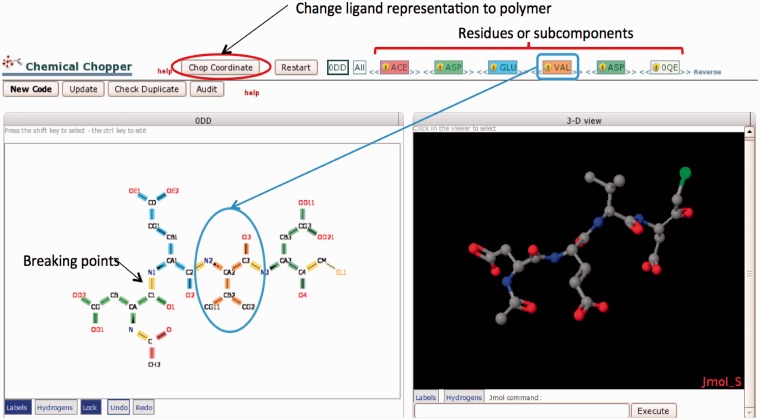
The Chopper Tool is used to break peptide-like inhibitors and antibiotics into individual polymeric residues or subcomponents following BIRD definitions (Step 2.4 in Figure 1). Edits are made in the 2D view (lower left); selected bonds (highlighted in yellow) can be marked to be ‘chopped’. The chopped residue or subcomponent is then searched against the CCD; results are color-coded and listed in the top bar. For example, VAL is colored coded in orange. The order of residues/subcomponents listed at the top of the page can be changed (by dragging the name) as needed to provide the appropriate sequence. The 3D view (right) displays the same components shown in the 2D view. When the subcomponents have been created and the sequence is in the correct order, the ‘Chop Coordinates’ button will change the molecule to a polymeric representation in the PDB entry according to the selected decomposition.

These peptide-like inhibitors and antibiotics are now represented in PDB entries as either polymers or large ligands with subcomponent (sequence) information. In the latter representation, the sequence information of the large ligand (list of subcomponents that constitute a large ligand) is also defined in the CCD. For example, the thiostrepton molecule (from PDB entry 1E9W) is represented in the BIRD as a polymer with a sequence of 19 peptide-like residues, whereas the molecule lisinopril (from PDB entry 1O86) is represented in the CCD as a large ligand with ligand code LPR consisting of three subcomponents (CLT, LYS and PRO). A ‘Chopper’ Tool (Figure 5) was created and added to the CCA Tool to facilitate creation of the peptide-like representation of these molecules (Figure 1, Step 2.4).

Deconstruction or bond-breaking of the larger molecule is performed using the Chopper Tool’s 2D and 3D viewing panels. Bond-breaking points are automatically selected and highlighted in yellow, and can be adjusted by the annotator (Figure 5, left panel). The molecule is then separated into its constituent peptide-like residues or subcomponents. These subcomponents are color-coded in the 2D chemical diagram. Importantly, the atoms leaving on bond formation are added back to the residues to complete the subcomponent molecules in their free and neutral charge state (the –OH and –H are added by default). The resulting complete peptide-like residues or subcomponents are then compared against the CCD for matches. The resulting matches are displayed in the top bar sequence representation. The sequence is displayed from the N-terminus to the C-terminus by default; however, the order of the residues can be manipulated or reversed in the case of cyclic peptides (Figure 5).

As in the CCE, the Chopper Tool is equipped with a full spectrum of chemical editing functions, so that each residue or subcomponent can be further reviewed, edited and added to the CCD. If a polymeric representation is chosen, all changes including nomenclature, representation and sequence order are then applied to the coordinates using the ‘Chop Coordinates’ button (Figure 5). The polymer sequence for a polymeric representation or the subcomponent sequence for a single molecule representation will appear in the PDB entry.

## CCD ID assignment and nomenclature standardization

Once all of the chemical components are defined and assigned, the coordinates for the PDB entry are updated to include the correct CCD ID, standardized atom nomenclature matching the CCD definition and chemical name and formula (Figure 1, Step 2.5).

Additional ligand checking has been added to the D&A pipeline in the Validation module. Cross-validation checking against the Cambridge Structural Database (CSD) (14) is run using CSD’s library of molecular geometry called Mogul. Bond lengths, bond angles, acyclic torsions and isolated rings are assessed by comparison with preferred molecular geometries derived from high-quality small-molecule structures in the CSD.

## Computational infrastructure

The CCA Tool is built as an independent module, but also with the ability to be integrated with the D&A workflow manager system for automation of the full annotation pipeline. This module has been implemented as a three-tiered Web-based client/server application. It uses jQuery/JavaScript/Cascading Style Sheets technologies at the front end, Apache application server as the middle tier and C++ applications at the back-end. The back-end C++ applications are wrapped by a Python application programming interface that is handled by the Apache application server.

Chemical component searching and processing is performed on the back-end by C++ programs, some of which are shared with the Ligand Expo Web site (http://ligand-expo.rcsb.org) that provides public access to the contents of the CCD.

Many chemical features are calculated using open-source and commercial chemical tools: Open Babel (15) for adding hydrogen and bond perception, CACTVS (16) for stereochemical assignment from 3D coordinates and SMILES generation, CORINA (17) for computing idealized 3D coordinates [parameters are described in Chapter 12 of (18)], OpenEye (http://www.eyesopen.com) for 2D chemical structure depiction and SMILES, ACDLabs (http://www.acdlabs.com) for SMILES and IUPAC name, InChI (19) for InChI and InChiKey reference and VF library, a graph matching library that provides several algorithms for subgraph isomorphism of the chemical graphs generated during chemical matching process (20).

Information derived from the back-end ligand searching and processing is then used to dynamically populate HTML templates for review. Once the HTML markup is delivered to the Web browser, user interaction with the screen elements used for ligand searching and assignment is governed by various JavaScript/Cascading Style Sheets/AJAX mechanisms. These mechanisms are facilitated primarily by use of the jQuery JavaScript library (http://jquery.com).

The interface launched to edit an existing or create a new chemical definition runs in the browser and was developed in JavaScript. The main 2D chemical diagram view is based on the HTML5 canvas, does not require a particular device or plugin and is a complete re-implementation of a typical chemical diagram editor. In contrast with other chemical diagram editors available online (21), it uses asynchronous requests to utilize various chemo-informatics packages on the server side and integrates functionality of CACTVS, CORINA and OpenEye. The editor uses various open-source JavaScript libraries (e.g. jQuery, jqGrid and jQuery Windows Engine) and Jmol (http://jmol.sourceforge.net). It follows a Model-View-Controller design that allows users to interact with the ligand using the chemical diagram editor, Jmol, a tabular view (jqGrid) or a chemical format text view.

The editor and common chemical search developed for the overall tool are reused in the Chopper Tool to search, match and annotate subcomponents of large molecules. The Chopper Tool supports additional specific requirements such as color highlighting of subcomponents, and data-model support for atom references between the ligand and its subcomponents.

## Testing and implementation

The software framework has been developed in a test-driven manner that permits unit and modular testing of each key component. This allowed chemical component search and matching subsystem testing to be performed independent of the user interface and with a high degree of automation. A similar testing strategy has been employed for other functional components; moreover, all of the operations of the CCA Tool are executable components of a workflow automation system that provides another vehicle for instrumenting functional testing.

Following a period of both unit and integration testing, the CCA Tool was incorporated into the current data processing pipelines at all three wwPDB sites. To protect unreleased data, the system is secured by passwords and is accessible only via the wwPDB’s internal network. The Web-based interface is browser- and operation system-independent, giving users maximal flexibility.

This module integrates and communicates effectively with other modules through a workflow manager in the D&A system. As part of the integrated environment, the module saves the chemical component assignments and definitions, passes such information to the next module and updates the PDB entry accordingly.

The use of the CCA Tool in production for the processing of hundreds of structures has proven that it is reliable and stable. Stress testing has been performed using multiple simultaneous users and with structures that have tens to hundreds of ligands. The tool performed well during these tests. Benchmark performance testing was carried out by the following method: the same annotator processed the same PDB entry using both the legacy method and the new CCA Tool for efficiency comparison. The results recorded in this performance testing included the number of ligands present in a PDB entry, the loading time for subgraph search and result display and the overall ligand processing time per entry. Many entries containing a total of 92 instances were tested to obtain an average result.

Overall, benchmark study has shown significant improvements to CCA processing efficiency and accuracy in chemical component assignments and definitions. With the reduction of PDB entry processing time compared with the previous CCA process, annotation efficiency improved up to 70%.

Many of the features available in the CCA Tool, including the chemical subgraph search against the CCD, the 2D and 3D chemical comparison view and the ability to assign CCD IDs, are available for depositors submitting data as part of the new wwPDB D&A system. Depositing authors can also provide additional chemical information in this system to ensure proper component creation and verification during annotation.

## Conclusion

This CCA Tool is an important module in the new wwPDB D&A system for processing deposited PDB data. It extracts chemical components from the submitted PDB entry, searches for these components in the CCD and displays the top candidates for further analysis by the entry’s annotator. It then updates both the ligand definition and structural model as needed. For a new component, the CCA Tool provides a chemical editor to create a new definition. For larger molecules that require the creation of polymeric representation, the Chopper Tool breaks the ligand molecule into covalently linked monomers (polymers).
